# Geographic Variations in Test Reactivity for the Serological Diagnosis of Trypanosoma cruzi Infection

**DOI:** 10.1128/JCM.01062-21

**Published:** 2021-11-18

**Authors:** Carine Truyens, Eric Dumonteil, Jackeline Alger, Maria Luisa Cafferata, Alvaro Ciganda, Luz Gibbons, Claudia Herrera, Sergio Sosa-Estani, Pierre Buekens

**Affiliations:** a Laboratory of Parasitology, Faculty of Medicine, and ULB Center for Research in Immunology (UCRI), Université Libre de Bruxelles, Brussels, Belgium; b Department of Tropical Medicine, School of Public Health and Tropical Medicine, and Vector-Borne and Infectious Disease Research Center, Tulane University, New Orleans, Louisiana, USA; c Instituto de Enfermedades Infecciosas y Parasitología Antonio Vidal, Departamento de Laboratorio Clínico, Hospital Escuela, Facultad de Ciencias Médicas, UNAH, Tegucigalpa, Honduras; d Unidad de Investigación Clínica y Epidemiológica Montevideo (UNICEM), Montevideo, Uruguay; e Institute for Clinical Effectiveness and Health Policy, Buenos Aires, Argentina; f Drugs for Neglected Diseases initiative-Latin America, Rio de Janeiro, Brazil; g Epidemiology and Public Health Research Center (CIESP-CONICET), Buenos Aires, Argentina; h Department of Epidemiology, School of Public Health and Tropical Medicine, Tulane University, New Orleans, Louisiana, USA; Mayo Clinic

**Keywords:** diagnostic performance, ELISA, rapid test, reactivity, strain diversity, Chagas disease, diagnostics, serology

## Abstract

Chagas disease is a neglected disease caused by Trypanosoma cruzi parasites. Most diagnosis is based on serological tests, but the lack of a gold standard test complicates the measurement of test performance. To overcome this limitation, we used samples from a cohort of well-characterized T. cruzi-infected women to evaluate the reactivity of two rapid diagnostic tests and one enzyme-linked immunosorbent assay (ELISA). Our cohort was derived from a previous study on congenital transmission of T. cruzi and consisted of 481 blood/plasma samples from Argentina (*n* = 149), Honduras (*n* = 228), and Mexico (*n* = 104), with at least one positive T. cruzi PCR. Reactivity of the three tests ranged from 70.5% for the Wiener ELISA to 81.0% for the T-Detect and 90.4% for the Stat-Pak rapid tests. Test reactivity varied significantly among countries and was highest in Argentina and lowest in Mexico. When considering at least two reactive serological tests to confirm seropositivity, over 12% of T. cruzi infection cases from Argentina were missed by serological tests, over 21% in Honduras, and an alarming 72% in Mexico. Differences in test performance among countries were not due to differences in parasitemia, but differences in antibody levels against ELISA antigens were observed. Geographic differences in T. cruzi parasite strains as well as genetic differences among human populations both may contribute to the discrepancies in serological testing. Improvements in serological diagnostics for T. cruzi infections are critically needed to ensure an optimum identification of cases.

## INTRODUCTION

Chagas disease is a neglected tropical disease caused by the protozoan parasite Trypanosoma cruzi. It is transmitted principally by hematophagous triatomine bugs, although congenital and oral transmissions are gaining importance. Infection leads to chronic cardiac disease in 30 to 40% of patients, eventually causing cardiac failure (1). Digestive forms of the disease may also develop. The disease affects 6 to 10 million persons, mostly in the Americas, where vectorial transmission is endemic, and is associated with health care costs of over $24 billion (2).

The identification of infected patients is critical to ensure early treatment and care, as the efficacy of available drugs decreases when the infection becomes more chronic. It is also critical for the screening of blood donors, to avoid transfusion-associated transmission, and for the screening of pregnant women, to identify those at risk of congenital transmission. Finally, it is essential for global disease surveillance to evaluate disease burden. Since most infected persons are chronically infected, diagnosis is mostly based on serological tests that detect circulating antibodies against the parasite. Multiple tests have been developed over the years, often based on enzyme-linked immunosorbent assay (ELISA) platforms and multiple antigens, ranging from crude parasite lysates/extracts to purified antigens or selected recombinant proteins (3). Several rapid tests based on recombinant proteins and immunochromatographic platforms have also been developed (4). While most tests are thought to have high sensitivity and specificity, their actual performance may be overestimated (4–6). Thus, current recommendations require a positive reaction with two distinct serological tests for a confirmed diagnosis of T. cruzi infection. In case of discordance between the tests, a third test needs to be performed. This complicates diagnostic algorithms and can severely delay the identification of cases and their adequate care (7).

Furthermore, variations in test performance among regions and countries have been reported before (8), particularly in Mexico (9, 10). A recent assessment of test performance among U.S. blood donors indicated that for all tests evaluated, antibody reactivity and clinical sensitivity were lowest in donors from Mexico, intermediate in those from Central America, and highest in those from South America, although minimum sensitivity reached at least 82.6% (11, 12). In a previous study, we screened pregnant women for T. cruzi infection in a large multicentric study in Argentina, Honduras, and Mexico to assess congenital transmission (13). Among 28,145 women screened with two rapid tests, we identified 495 with at least one reactive rapid test, of which 347 (70%) were confirmed by ELISA. Again, discordant serology seemed to be more important in samples from Mexico than Argentina (13).

Nonetheless, as noted before (14), a key issue in assessing test performance is the lack of a gold standard for the serological diagnosis of T. cruzi infection, so that sensitivity of tests is usually determined using samples that are strongly reactive in multiple serological tests, and discordant samples are considered false positives and often discarded from analyses. To overcome this limitation, we aimed to measure the reactivity of commercial serological tests using a unique cohort of samples with at least one positive T. cruzi PCR and, thus, can be considered true infection cases.

## MATERIALS AND METHODS

### Cohort.

We used a cohort of maternal blood samples derived from a previous multicentric study aiming to evaluate the congenital transmission of T. cruzi in Argentina, Honduras, and Mexico (13). Mothers were recruited at delivery in one hospital in Argentina (Instituto de Maternidad y Ginecología Nuestra Señora de las Mercedes, Tucuman), two hospitals in Honduras (Hospital Enrique Aguilar Cerrato, La Esperanza, Intibucá, and Hospital Santa Bárbara Integrado, Santa Bárbara), and two hospitals in Mexico (Hospital Materno Infantil, Mérida, Yucatán, and Hospital General de Valladolid, Valladolid, Yucatán). Cord blood samples were initially screened by two rapid tests (T-Detect and Stat-Pak), and if one of the rapid tests was reactive, maternal blood was collected to assess maternal T. cruzi infection, as detailed in the study protocol (15). The cohort included here consisted of 481 maternal blood samples considered true T. cruzi infection cases, based on at least one positive T. cruzi PCR. For PCR testing, three PCR assays were performed: two conventional PCRs targeting nuclear satellite DNA (Sat. DNA PCR; TcZ1/TcZ2 primers) (16), parasite kinetoplast DNA (kDNA PCR; Tc121/Tc122 primers) (17), and a quantitative real-time PCR (qPCR) targeting nuclear satellite DNA (TcZ1-TcZ2 primers) (13). DNA was extracted from 300 μl of blood conserved in 6 M guanidine HCl, 0.2 M EDTA using an automated Maxwell RSC instrument (Promega Benelux, Leiden, The Netherland) as described previously (13). All DNA extractions and PCR assays were performed in a single laboratory in Brussels (Belgium). There were 149 samples from Argentina, 228 from Honduras, and 104 from Mexico (Table 1). The study was approved by the IRB committees of Tulane University and all other participating institutions in Argentina, Honduras, and Mexico.

**TABLE 1:** Cohort of PCR-positive maternal blood samples^*a*^

Test	Argentina (*n* = 149)	Honduras (*n* = 228)	Mexico (*n* = 104)	Total (*n* = 481)
kDNA PCR	81/149 (54.4)	131/228 (57.5)	27/104 (25.9)	239/481 (49.7)
Sat. DNA PCR	94/149 (63.1)	127/228 (55.7)	40/104 (38.5)	261/481 (54.3)
kDNA or Sat. DNA PCR	120/149 (80.5)	163/228 (71.5)	53/104 (51.0)	336/481 (69.9)
Sat. DNA qPCR	147/149 (98.7)	226/228 (99.1)	100/104 (96.5)	473/481 (98.3)

a Data are presented as number of positive samples/total samples tested (% positive).

### Serological tests. (i) Rapid tests.

Following written consent, venous whole blood was collected from women soon after birth in EDTA Vacutainer tubes. Within 6 h of sample collection, Stat-Pak (Chembio, Medford, NY) and *Trypanosoma* Detect (T-Detect; InBios, Seattle, WA) rapid diagnostic tests were performed on blood-EDTA by following the manufacturer’s instructions in each hospital laboratory. Test results were read by laboratory technicians at 15 and 10 min, respectively, and photographed for a subsequent second confirmatory reading by another technician, allowing for quality control. Stat-Pak contains antigens B13, H49, and 1F8, while T-Detect is based on a fusion peptide made up of portions of antigens 1, 30, 36, SAPA, Kmp-11, and TcF.

### (ii) ELISA.

Plasma was prepared from the blood-EDTA samples and tested by ELISA (Chagatest ELISA recombinant V3.0; Wiener, Rosario, Argentina) according to the manufacturer’s instructions. Plates were read at 450 nm. Two positive and three negative controls were included on each plate. The cutoff corresponded to the mean optical density at 450 nm (OD_450_) of negative controls + 0.300. The gray zone giving indeterminate results corresponded to the cutoff ±10%. Samples with an OD less than the cutoff minus 10% were interpreted as negative, and those with an OD greater than the cutoff plus 10% were positive. A random subset of 10% of the samples was tested again by following the same protocol in a reference laboratory for quality control. Wiener’s Chagatest consists of antigens 1, 2, 13, 30, 36, and SAPA.

### Statistical analyses.

We calculated the reactivity of the serological tests for identifying T. cruzi infection among our cohort of maternal samples with at least one positive T. cruzi PCR. This is similar to the sensitivity of the tests, but because the initial enrollment of women was based on screening cord blood samples with the same rapid tests, complete independence could not be ascertained for our cohort of women with positive T. cruzi PCR. Proportions among countries were compared using chi-square tests, and 95% confidence intervals (CI) were calculated. Histogram distributions of parasitemia, measured by qPCR, were constructed to compare parasite levels among women from each country. Distributions were fitted to normal distributions. Similarly, distributions of antibody levels, measured as ELISA absorbance readings, were compared among countries. Distributions were fitted to mixtures of 2 to 3 normal distributions. All analyses were performed with the software JMP (SAS Institute Inc., Cary, NC).

## RESULTS

We measured the performance of the three serological tests (one ELISA and two rapid tests) carried out on maternal blood samples among samples from a reference cohort of T. cruzi-infected women, based on at least one positive PCR test (Table 1). The reactivity of the tests was compared among the three countries (Table 2). Stat-Pak rapid test presented the highest overall reactivity (90.4%; 95% confidence interval [CI], 87.5 to 92.7), followed by T-Detect (81.0%; 95% CI, 76.9 to 83.9) and ELISA (70.5%; 95% CI, 66.2 to 73.4).

**TABLE 2:** Reactivity of serodiagnostic tests

Test	Argentina	Honduras	Mexico	Total
Stat-Pak^*a*^	145/149 (97.3)	219/228 (96.1)	70/104 (67.3)	435/481 (90.4)
T-Detect^*a*^	129/149 (86.6)	189/228 (82.9)	69/104 (66.4)	388/481 (81.0)
ELISA^*a*^	131/149 (87.9)	179/228 (78.5)	29/104 (27.9)	339/481 (70.5)

a Statistically significant difference among countries. Data are presented as number of reactive samples/total samples tested (% reactive).

Furthermore, there were marked differences in test reactivity among countries. Indeed, Stat-Pak tests presented a high reactivity in Argentina (97.3%; 95% CI, 93.3 to 98.9) and Honduras (96.1%; 95% CI, 92.7 to 97.9) but not in Mexico, where reactivity was 67.3% (95% CI, 57.8 to 75.6) (χ^2^ = 63.7; degrees of freedom [df] = 2; *P* < 0.0001). The reactivity of T-Detect was somewhat lower, reaching 86.6% (95% CI, 80.2 to 91.1) in Argentina and 82.9% (95% CI, 77.5 to 87.2) in Honduras, but it was also significantly lower in Mexico (66.4%; 95% CI, 56.7 to 74.7) (χ^2^ = 16.2; df = 2; *P* = 0.0003). Finally, the reactivity of the ELISA was 87.9% (95% CI, 81.7 to 92.2) in Argentina but only 78.5% (95% CI, 72.3 to 83.3) in Honduras and 27.9% (95% CI, 20.2 to 37.2) in Mexico (χ^2^ = 117.8; df = 3; *P* < 0.0001).

Because current recommendations require two reactive serological tests to confirm seropositivity, we further examined the proportion of true T. cruzi infections that were detected by serology based on different combinations of serological tests (Table 3). Considering different combinations of rapid tests plus ELISA, the best performance was obtained for a combination of both rapid tests and ELISA, which allowed identifying 70.5% of true T. cruzi infections (95% CI, 66.2 to 74.4). However, performance again was significantly different among countries. In Argentina, up to 87.9% (95% CI, 81.7 to 92.2) of T. cruzi infections and 78.5% (95% CI, 72.7 to 83.3) in Honduras were diagnosed. Strikingly, only 27.9% of T. cruzi infections were detected by serology in Mexico (95% CI, 20.2 to 37.2). Thus, many cases of maternal infection were missed by serology (Table 3).

**TABLE 3:** Serodiagnosis of Trypanosoma cruzi infection with multiple tests

Test	Argentina	Honduras	Mexico	Total
SP + ELISA^*a*^	128/148 (86.5)	175/228 (76.8)	27/104 (26.0)	330/480 (68.8)
TD + ELISA^*a*^	121/148 (81.8)	177/228 (77.6)	23/104 (22.1)	321/480 (66.9)
Any rapid test + ELISA^*a*^	131/149 (87.9)	179/228 (78.5)	29/104 (27.9)	339/481 (70.5)
2 rapid tests^*a*^	125/149 (83.9)	180/228 (79.0)	35/104 (33.7)	341/481 (70.8)

a Statistically significant difference among countries. Data are presented as number of T. cruzi infected samples/total samples tested (% infected).

Interestingly, the combination of two rapid tests performed in a manner very similar to that of combinations of rapid tests plus ELISA and allowed the identification of 70.8% of all T. cruzi infections (95% CI, 66.7 to 74.8) (versus 70.5%; 95% CI, 66.2 to 74.4) (Table 3). There was, however, a difference among countries (χ^2^ = 82.8; df = 2; *P* < 0.0001). In Argentina, 83.9% (95% CI, 77.1 to 88.9) of T. cruzi infections were diagnosed with only two rapid tests, 79.0% (95% CI, 73.2 to 83.7) in Honduras and only up to 33.7% (95% CI, 25.3 to 43.2) in Mexico.

**FIG 1:**
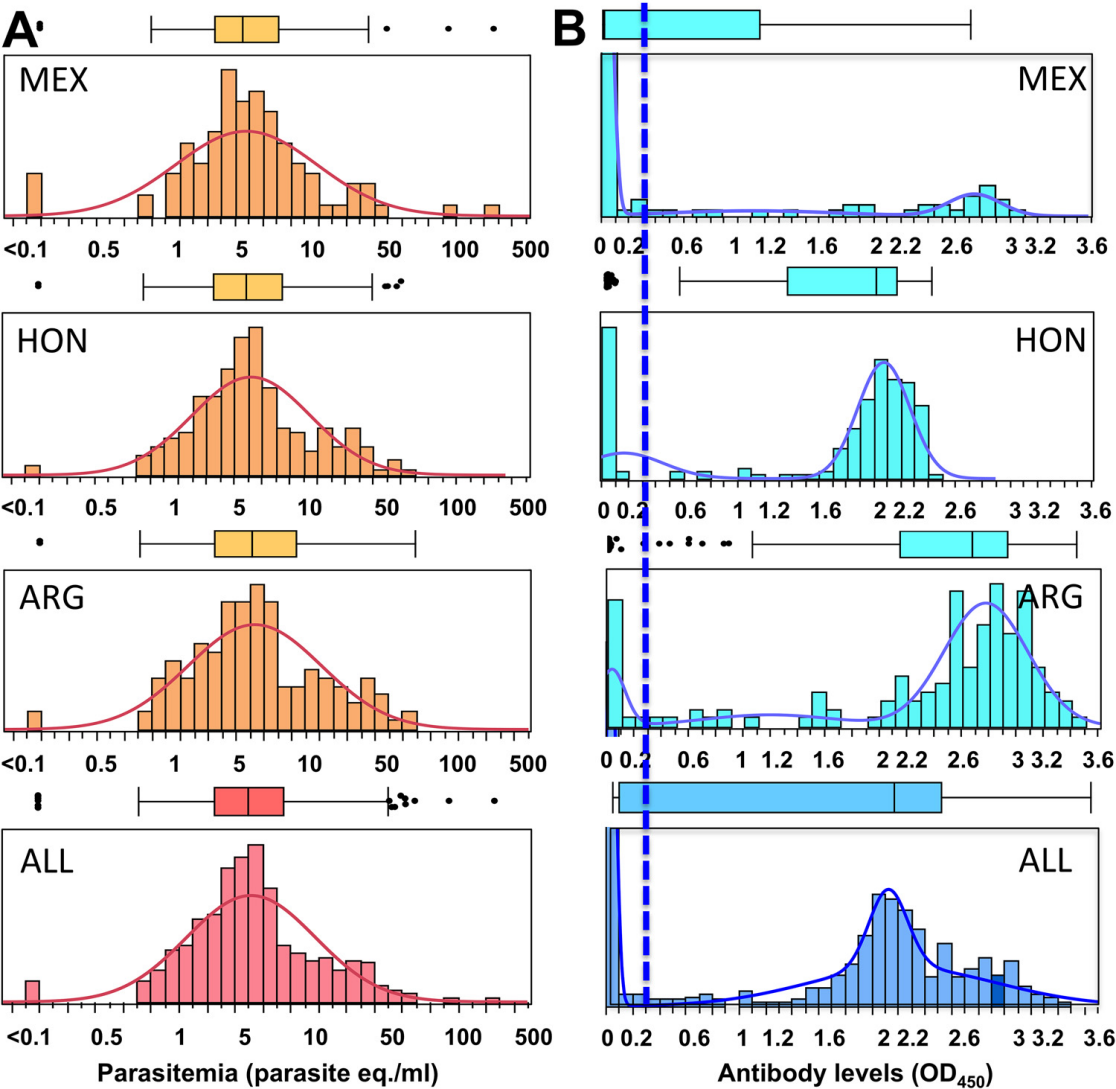
Histogram distributions of parasitemia and antibody levels per country. (A) Parasitemia levels were measured by qPCR and log transformed. Distributions were fitted to normal curves. Parasite levels and their distributions were very similar among the three countries (6.3 ± 8.2, 5.4 ± 6.5, and 7.0 ± 19.5 parasite equivalents/ml for samples from Argentina, Honduras, and Mexico, respectively; ANOVA *F* = 0.77; *P* = 0.46). Box plots are shown above each histogram. (B) Antibody levels were measured by ELISA and measured as OD_450_. Distributions were fitted to mixtures of 2 to 3 normal distributions. The vertical dotted line indicates the cutoff value of the ELISA (OD_450_ = 0.300). Box plots are also shown above each histogram. MEX, Mexico; HON, Honduras; ARG, Argentina; ALL, combined total.

We then evaluated possible explanations for the differences in serological test performance among countries. We analyzed parasite levels in the blood samples, as measured by qPCR, to test the hypothesis that differences in parasitemia cause differences in antibody responses. We also analyzed antibody levels, as measured by OD_450_ readings of the ELISA, to assess potential differences in antigen-specific IgGs. Parasite levels were very similar among samples from the three countries (means ± standard deviations, 6.3 ± 8.2, 5.4 ± 6.5, and 7.0 ± 19.5 parasite equivalents/ml for samples from Argentina, Honduras, and Mexico, respectively; *F* = 0.77 and *P* = 0.46 by analysis of variance [ANOVA]) and followed similar distributions (Fig. 1A). There also was no correlation between parasite and antibody levels in any of the countries (*P* = 0.25, *P* = 0.92, and *P* = 0.69 for Argentina, Honduras, and Mexico, respectively). Thus, differences in serological test performance were not due to differences in parasitemia.

On the other hand, antibody levels against ELISA antigens and their distributions were very different among the three countries (Fig. 1B). In Argentina, high antibody levels (OD_450_ around 2.8) were detected in most samples, with only a few reactive samples close to the cutoff value and a few seronegatives among the cohort of PCR-positive women. In Honduras, antibody levels were lower than those in Argentina (around 2.0), but most samples remained seropositive. In Mexico, only a small proportion of T. cruzi-infected women had antibody levels as high as those in Argentina (OD_450_ around 2.8), many had low antibody levels (OD_450_ between 0.6 and 2.0), and most were completely seronegative. These data clearly indicate that there were important differences in antibody levels against the ELISA antigens among women from the different countries.

## DISCUSSION

Understanding the performance of serological tests is critical for optimal identification of infected patients and for Chagas disease surveillance. However, it is hindered by a lack of a gold standard for serological diagnostic (18). To overcome this limitation, we used here a cohort of 481 true T. cruzi-infected samples, as determined by PCR for the parasite, derived from a multicountry study, and measured the reactivity of two rapid diagnostic tests and one ELISA, all based on different sets of recombinant antigens (or fusion proteins covering several antigens).

The overall reactivity of the three tests was rather low, ranging from 70.5% to 90.4%. Most remarkably, there were important differences in test reactivity among countries, as tests performed best in Argentina and poorly in Mexico. It is also interesting that both rapid tests had a better reactivity than the ELISA, but this may be an overestimation due to the enrollment of the women based on a reactive rapid test in cord blood samples from their newborns.

When considering combinations of serological tests, as currently required to establish seropositivity of a patient, the highest reactivity was obtained when considering any one of the two rapid tests plus ELISA, reaching 70.5%. Strikingly, even in Argentina, where tests performed the best, over 12% of T. cruzi infection cases from this cohort were missed by serological tests. Even more preoccupying, over 21% of infections were missed in Honduras and an alarming 72% of infections in Mexico. Considering that these commercial tests are among the most widely used in Mexico (19), current estimates of Chagas disease burden in this country may be severely underestimated. Discordant serology and underdiagnosis should also be of growing concern in nonendemic regions, where patients may be left with inconclusive test results that delay or prevent their access to drug treatment and care (20).

Interestingly, the combination of the two rapid tests had a reactivity very similar to that of the combination of rapid tests plus ELISA. This is in agreement with previous studies in Bolivia and Argentina, where the use of two rapid tests was proposed as a novel strategy for point-of-care testing to accelerate patient identification and access to drug treatment (21, 22). This seems to be a promising strategy to facilitate access to diagnostics, which is one of the first key barriers that needs to be overcome to improve health care for Chagasic patients (23, 24). However, due to geographic differences in test performances, the usefulness of the rapid tests (as for any other tests) needs to be carefully evaluated in populations from different geographic origins, as well as different clinical status, before widespread use.

Understanding the underlying mechanisms of differences in test reactivity would be critical, as it may lead to test improvement. Our first hypothesis, that differences in parasitemia levels may be reflected in low antibody levels (below the detection threshold of serological tests), was clearly discarded by the analysis of parasite levels by qPCR. Indeed, patients from all three countries presented very similar levels of parasitemia, confirming earlier results on a different subset of samples (13). Thus, there is a discrepancy between parasite levels and patient’s antibody responses. Further analysis of antibody levels as measured by OD_450_ levels in the ELISA revealed clear differences among countries. While infected women from Argentina presented high antibody levels specific for test antigens, women from Honduras had lower antibody levels. In Mexico, very few women had high antibody levels, and most of them had no antibodies against test antigens. Such differences in antibody levels against test antigens are similar to those reported in U.S. blood donors coming from different countries in Latin America (12) and could be due to differences in immune response (host genetic diversity) and/or difference in antigens (parasite diversity). Interestingly, the recently described reference serum standards derived from samples from Mexico, thought to be infected with TcI, and samples from Brazil and Argentina, thought to be infected with TcII, seem to present some differences in OD reading between the two pools of samples (25), suggesting different antibody levels against ELISA antigens among these countries.

However, an initial analysis of parasite genetic diversity among 105 blood samples from our cohort of pregnant women showed a similar distribution of parasite discrete typing units (DTUs) among the three countries, with a large predominance of non-TcI DTUs followed by mixed infections with TcI and non-TcI DTUs and a very low proportion of TcI infections, including in Mexico (26). Nonetheless, sequence analysis revealed a significant difference between specific sequence haplotypes and seropositivity in spite of the short length of the sequence analyzed (26). Thus, the genetic diversity of T. cruzi parasites may contribute to geographic differences in serological test performance. Analysis of T. cruzi genotypes infecting dogs in southern Louisiana also suggested that these are associated with discordant serology (27). Several studies have previously shown that the use of parasite antigens derived from local strains improves test performance (9, 10, 28, 29).

While T. cruzi genetic diversity has been known for a long time (30, 31) and has led to its current division into seven DTUs (32), the extent of this diversity within DTUs and across geographic regions is only beginning to be uncovered (33–35). Indeed, several antigens used in the rapid tests and ELISA evaluated here are much less conserved among parasite strains than initially believed, which may impact their usefulness in diagnostic tests (35). Different mixtures of antigens, and their respective level of conservation among parasite strains, may explain the differences in test performance we observed. Thus, it is critical to better understand the geographic distribution of T. cruzi strain and haplotype diversity to ensure that test antigens are sufficiently conserved in the different regions. Additional sequence information on the T. cruzi strains infecting women from our cohort would also allow us to further refine how parasite genetic diversity is associated with diagnostic test performance. In addition, assays based on whole parasite antigens/extracts may be more sensitive, as they detect a broader range of antibodies produced during the infection, but they also may be less specific due to cross-reactions.

Host genetic differences, such as HLA class I and II alleles and single-nucleotide polymorphisms in coding and noncoding regions of cytokines and cytokine receptors, may also further contribute to differences in antibody responses among countries (36, 37). Indeed, there are important differences in human leukocyte antigen (HLA) frequency between populations from Argentina and Mexico (http://www.allelefrequencies.net) that may result in different antigen recognition patterns among patients from these countries. In line with this, the prevalence of some protective alleles against infectious diseases of genes coding for HLA molecules or cytokines has been described to be very different in the Mexican population than other populations (38, 39). Thus, it would be of interest to evaluate such features in our cohort of infected women to assess potential associations with their immune response and serological test performance.

In conclusion, we analyzed the reactivity of two rapid diagnostic tests and one ELISA among a cohort of T. cruzi-infected women. The overall reactivity of the three tests was rather low, and there were important differences in test reactivity among countries. Over 12% of T. cruzi infection cases from Argentina were missed by serological tests, over 21% in Honduras, and an alarming 72% in Mexico. Differences in test performance among countries were not due to differences in parasitemia, but differences in antibody levels against ELISA antigens were clearly observed. Geographic differences in T. cruzi parasite strains as well as in HLA frequencies among populations both may contribute to these discrepancies in serological testing. It is critical to improve serological diagnostics for T. cruzi infections to ensure an optimum identification of cases and timely access to treatment.
